# A case of pleural effusion caused by *Mycobacterium fortuitum* and *Mycobacterium mageritense* coinfection

**DOI:** 10.1186/s12879-019-4366-8

**Published:** 2019-08-15

**Authors:** Ryosuke Hirabayashi, Atsushi Nakagawa, Hiroshi Takegawa, Keisuke Tomii

**Affiliations:** 1 Department of Respiratory Medicine, Kobe City Medical Center General Hospital, 2-1-1 Minatojima-minamimachi, Chuo-ku, Kobe, Hyogo 650-0047 Japan; 2 Department of Laboratory Medicine, Kobe City Nishi-Kobe Medical Center, 5-7-1 Kouji-dai, Nishi-ku, Kobe, Hyogo 651-2273 Japan

**Keywords:** Coinfection, *M. fortuitum*, *M. mageritense*, Nontuberculous mycobacteria, Pleural effusion

## Abstract

**Background:**

Non-tuberculous mycobacteria cause chronic pulmonary infection, but pleuritis and pleural effusion are rarely associated with infection with non-tuberculous mycobacteria, especially rapid-growing mycobacteria.

**Case presentation:**

A 68-year-old woman with rheumatoid arthritis who was using prednisone, azathioprine, and certolizumab pegol presented complaining of fever, dry cough, and night sweats for the past 2 weeks. Chest examination revealed bilateral opacity that was more pronounced on her right side. Bronchoalveolar lavage fluid and pleural effusion fluid were obtained, and revealed coinfection with *Mycobacterium fortuitum* and *Mycobacterium mageritense*. Imipenem/cilastatin, levofloxacin, and minocycline were prescribed for 6 months, and the patient was well and asymptomatic for the subsequent 6 months.

**Conclusions:**

This is the first case report describing pleural effusion associated with coinfection with two different mycobacterial species. If the species cannot be identified, the possibility of mycobacterial coinfection should be considered.

## Background

Non-tuberculous mycobacteria (NTM) are common organisms in the environment, and they can cause chronic pulmonary infection. As a group of NTM, rapid-growing mycobacteria (RGM) contain *Mycobacterium chelonae*, *M. fortuitum* and *M. mageritense* [1]. The most common sites of infection with these mycobacteria are pulmonary tissues, skin, bone, and soft tissue.

There are several reports of coinfection with different species of myobacteria. However, NTM infections rarely cause pleural effusion [1] and there are no reports of NTM coinfection associated with pleural effusion. Herein we report a case of pleural effusion caused by *M. fortuitum* and *M. mageritense* coinfection, which was identified from pleural effusion culture and bronchoalveolar lavage (BAL) fluid.

## Case presentation

A 68-year-old woman with rheumatoid arthritis was admitted to our hospital complaining of fever, dry cough, and night sweats for the past 2 weeks. She had been diagnosed with rheumatoid arthritis 9 years prior. She had been taking 5 mg/day prednisone and azathioprine, and she had started taking certolizumab pegol from 6 months prior to the current admission.

She reported no malaise, hemoptysis, or weight loss. Other than the aforementioned factors she had no past medical history, including interstitial lung disease or tuberculosis, she was not taking any other medications, and she had no pets. She had never smoked, and she worked as a pharmacist.

**Fig. 1:**
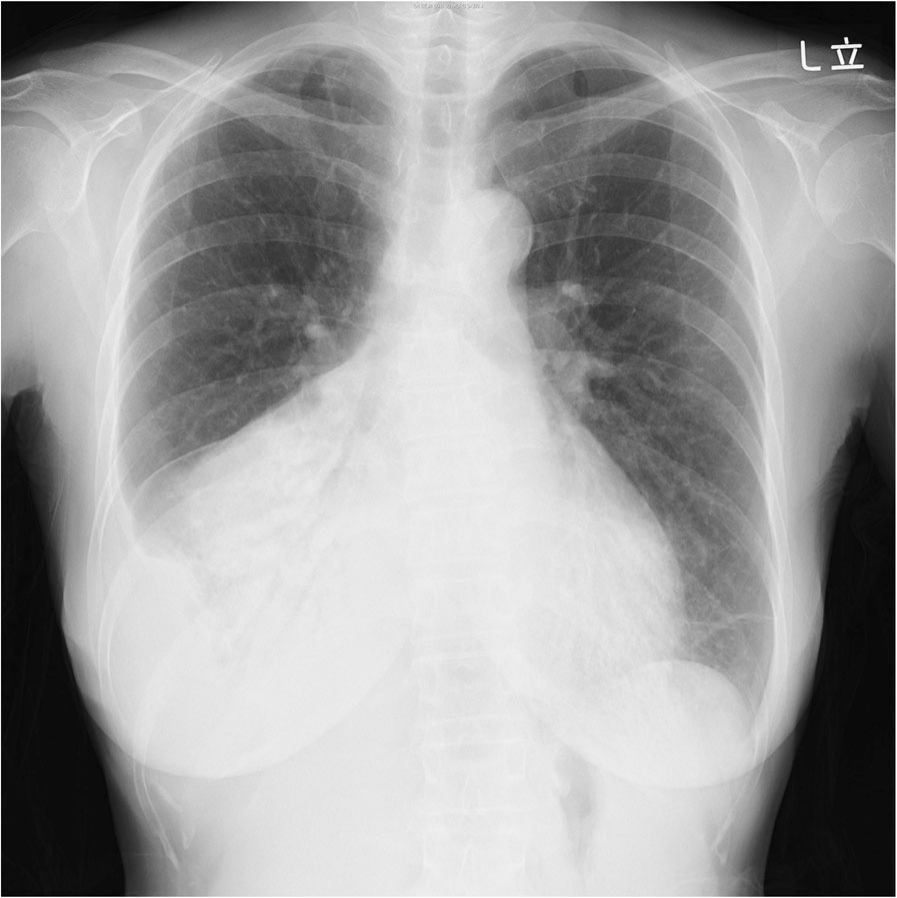
Chest x-ray image on admission

On admission her body temperature was 38.2 °C but other vital signs were not remarkable. Chest x-ray revealed bilateral opacity that was more pronounced on her right side (Fig. 1). Her white blood cell count was 11,700 μL^− 1^, hemoglobin was 10.0 g dL^− 1^, and C-reactive protein was 17.12 mg/dL. An HIV assay for HIV-1 and HIV-2 was negative, as was an interferon gamma release assay.

**Fig. 2:**
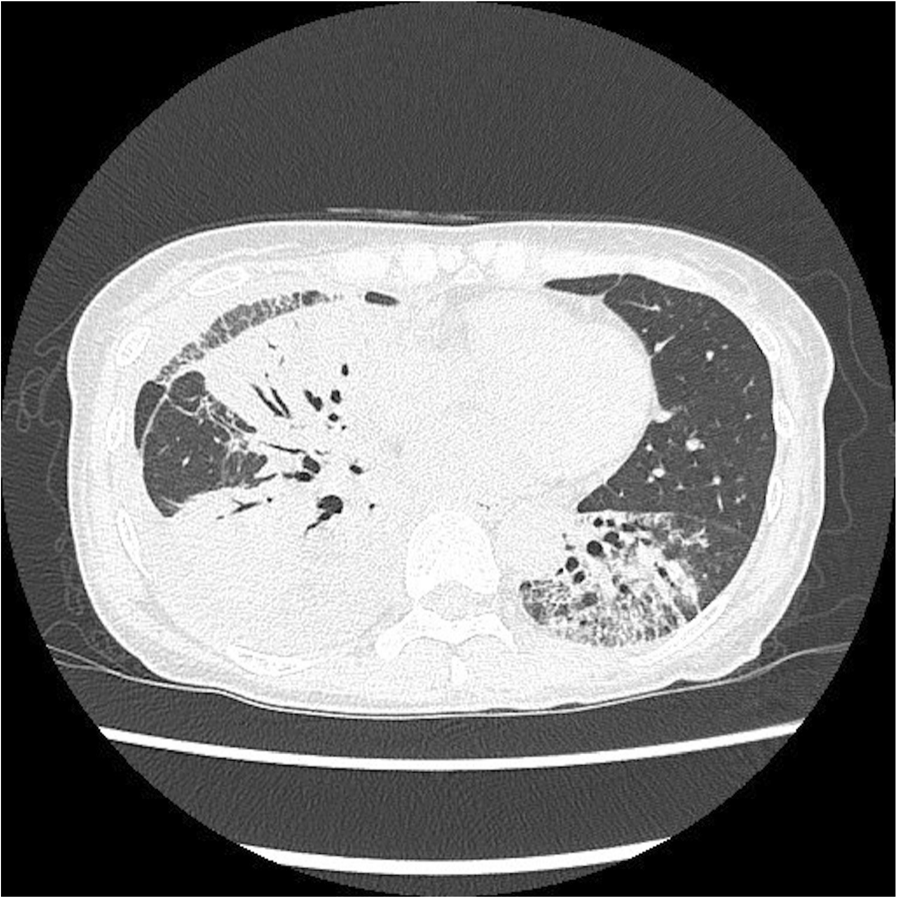
Chest CT image on admission

Chest computed tomography (CT) depicted bilateral opacity on her lower lung and pleural effusion on her right thoracic cavity (Fig. 2). Thoracentesis was performed to obtain 150 mL of pleural effusion fluid from the right side. The white blood cell count of that fluid was 12,700/μL^− 1^ (22.7% lymphocytes), and it contained 5.4 g protein/dL^− 1^, 2.8 g albumin/dL^− 1^, and 1503 U lactate dehydrogenase/L^− 1^.

BAL fluid and pleural fluid culture were performed. Acid-fast bacilli were detected by Ziehl-Neelsen staining but the species could not identified using a DNA-DNA hybridization method or the matrix-assisted laser desorption/ionization time-of-flight mass spectrometry (MALDI-TOF MS). The drug susceptibility testing revealed resistance to amikacin (minimum inhibitory concentration > 32 μg/mL), but reexamination for microbial identification and the drug susceptibility testing suggested susceptibility to amikacin. To investigate contamination or mycobacterial coinfection, growth enhancement on Middlebrook 7H11C agar plate testing was performed. Two types of colonies were developed from both BAL and pleural fluid culture, and the causative microorganisms were identified as *M. fortuitum* and *M. mageritense* by MALDI-TOF MS—with each of the hsp65 and rpoB region sequences respectively exhibiting 100 and 99.0% matching with *M. fortuitum*, and 98.4 and 99.0% matching with *M. mageritense*.

**Fig. 3:**
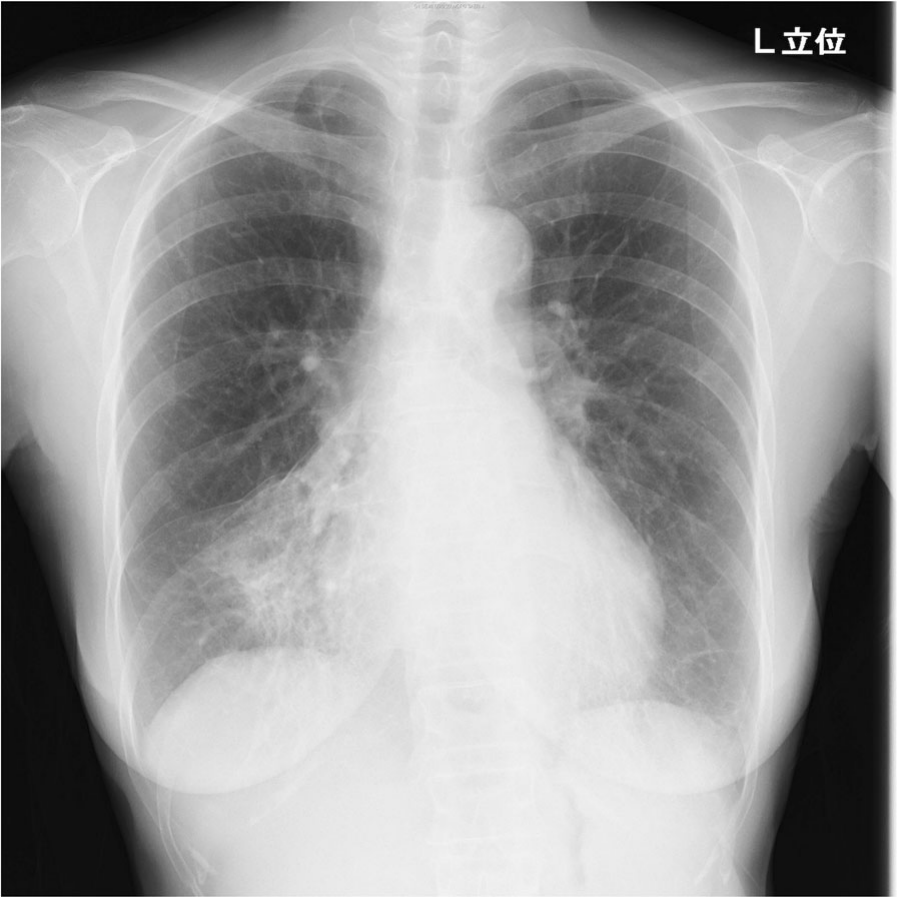
Chest x-ray image after the treatment

**Fig. 4:**
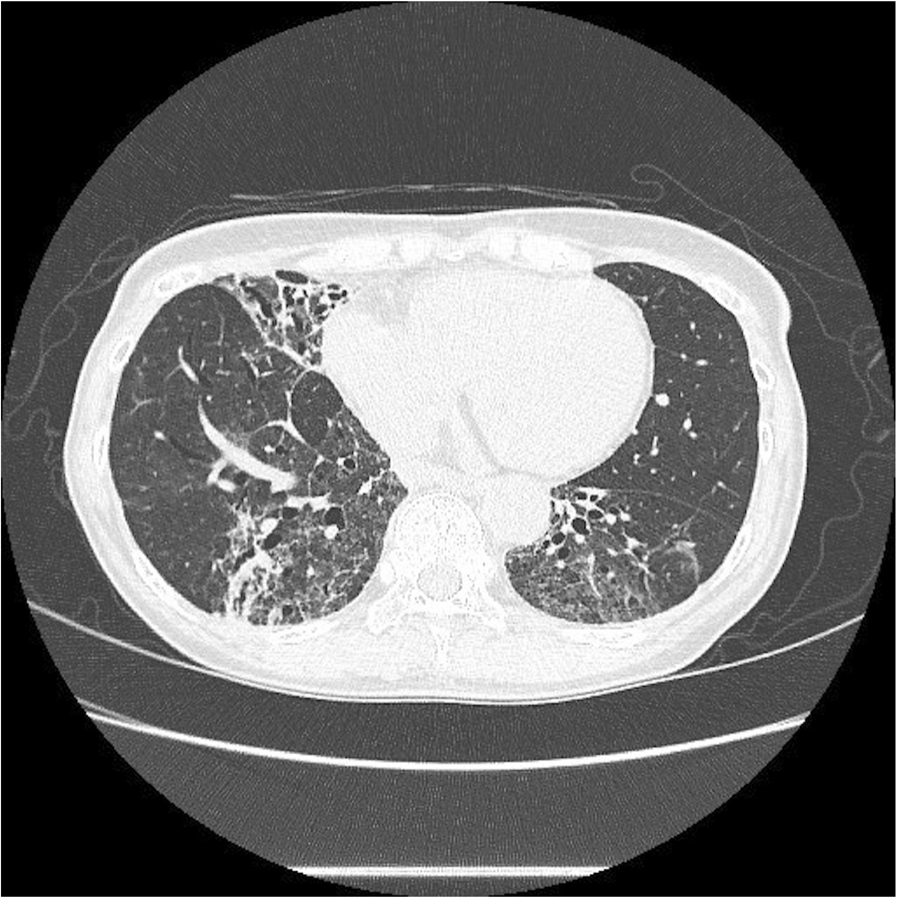
Chest CT image after the treatment

Eight weeks of treatment with imipenem/cilastatin (500 mg every 6 h intravenously), minocycline (100 mg orally twice a day), and levofloxacin (500 mg orally once a day) were initiated, followed by 4 months maintenance therapy by minocycline and levofloxacin. After the maintenance therapy, Her symptoms and bilateral pulmonary opacity were improved (Figs. 3 and 4). Now we follow her for 2 years, her sputum culture is still negative and her chest x-ray is still clear.

## Discussion and conclusion

This is the first report of pleural effusion caused by two species of RGM coinfection. There are several reports of pulmonary infection or pleural effusion caused by the coinfection of *mycobacteria*, which include NTM and *Mycobacterium tuberculosis* complex (MTC) coinfection or different subspecies of *M. avium* complex coinfection. In cases of pulmonary infection however, it is difficult to prove coinfection from sputum because it is easily colonized by NTM, and often it is difficult to isolate or identify the species involved. Aseptic collection of body fluid such as pleural effusion via needle aspiration is better for the verification of NTM coinfection [1], but NTM rarely causes pleural effusion than MTC. In the present case NTM coinfection was verified via pleural effusion and BAL. there are no previous reports of coinfection with nontuberculous mycobacteria in pleural effusions and this is the first report about it.

Neither DNA-DNA hybridization nor MALDI-TOF MS identified the species, and the aforementioned contradictory results of drug susceptibility testing were obtained twice. MALDI-TOF MS is one of the recommended molecular methods for identifying NTM [2], but the method is limited with regard to specificity in this respect (52.8–98.6%) [3, 4]. If the species cannot be identified, mycobacterial coinfection should be considered.

In conclusion, this report described a case of pleural effusion caused by *M. fortuitum* and *M. mageritense* coinfection. In cases of mycobacterial infection where microbiological testing cannot identify the species involved, coinfection should be considered and more extensive investigation to determine the species should be undertaken.

## Data Availability

The datasets used or analysed during the current study are available from the corresponding author on reasonable request.

## Abbreviations

BAL: Bronchoalveolar lavage
MALDI-TOF MS: Matrix-assisted laser desorption/ionization time-of-flight mass spectrometry
MTC: *Mycobacterium tuberculosis* complex
NTM: Non-tuberculosis mycobacteria
RGM: Rapid-growing mycobacteria
